# Unravelling the experiences of incarcerated individuals living with HIV on ART: a qualitative study in Ghanaian prisons

**DOI:** 10.1108/IJOPH-06-2023-0031

**Published:** 2024-01-19

**Authors:** Susanna Aba Abraham, Obed Cudjoe, Yvonne Ayerki Nartey, Elizabeth Agyare, Francis Annor, Benedict Osei Tawiah, Matilda Nyampong, Kwadwo Koduah Owusu, Marijanatu Abdulai, Stephen Ayisi Addo, Dorcas Obiri-Yeboah

**Affiliations:** Department of Adult Health, School of Nursing and Midwifery, University of Cape Coast, Cape Coast, Ghana; Department of Medical Laboratory Science, University of Cape Coast College of Health and Allied Sciences, Cape Coast, Ghana; Department of Internal Medicine, Cape Coast Teaching Hospital, Cape Coast, Ghana; Clinical Microbiology/Public Health Unit, Cape Coast Teaching Hospital, Cape Coast, Ghana and National HIV/AIDS Control Programme, Accra, Ghana; Directorate of Research Innovation and Consultancy, University of Cape Coast, Cape Coast, Ghana; Nsawam Medium Security Prisons, Ghana Prisons Service, Nsawam, Ghana; Headquarters, Ghana Prisons Service, Nsawam, Ghana; National AIDS/STI control Programme, Accra, Ghana; National HIV/AIDS Control Programme, Accra, Ghana; Department of Microbiology and Immunology, University of Cape Coast College of Health and Allied Sciences, Cape Coast, Ghana; Directorate of Research Innovation and Consultancy, University of Cape Coast, Cape Coast, Ghana and Clinical Microbiology/Public Health Unit, Cape Coast Teaching Hospital, Cape Coast, Ghana

**Keywords:** HIV/AIDS, Incarcerated individuals, Experiences, HIV service delivery, Prison service

## Abstract

**Purpose:**

The Joint United Nations Programme on HIV/AIDS (UNAIDS) goal to end the acquired immunodeficiency syndrome (AIDS) epidemic as a public health threat by 2030 emphasises the importance of leaving no one behind. To determine progress towards the elimination goal in Ghana, an in-depth understanding of human immunodeficiency virus (HIV) care from the perspective of vulnerable populations such as persons living with HIV in incarceration is necessary. This study aims to explore the experiences of incarcerated individuals living with HIV (ILHIV) and on antiretroviral therapy (ART) in selected Ghanaian prisons to help inform policy.

**Design/methodology/approach:**

The study adopted a qualitative approach involving in-depth interviews with 16 purposively selected ILHIV on ART from purposively selected prisons. Interviews were conducted between October and December 2022. Thematic analysis was performed using the ATLAS.Ti software.

**Findings:**

Three themes were generated from the analysis: waking up to a positive HIV status; living with HIV a day at a time; and being my brother’s keeper: preventing HIV transmission. All participants underwent HIV screening at the various prisons. ILHIV also had access to ART although those on remand had challenges with refills. Stigma perpetuated by incarcerated individuals against those with HIV existed, and experiences of inadequate nutrition among incarcerated individuals on ART were reported. Opportunities to improve the experiences of the ILHIV are required to improve care and reduce morbidity and mortality.

**Originality/value:**

Through first-hand experiences from ILHIV in prisons, this study provides the perception of incarcerated individuals on HIV care in prisons. The insights gained from this study can contribute to the development of targeted interventions and strategies to improve HIV care and support for incarcerated individuals.

## Introduction

Despite the significant advancements made in the prevention, early detection and treatment of human immunodeficiency virus (HIV) infection over the past four decades globally, HIV remains a disease of public health importance, especially in low- and middle-income countries and continues to pose threats to the health systems (CDC, 2023; WHO, 2023). By the end of 2021, approximately 38.4 million people across the globe were living with HIV and an estimated 650,000 people died from HIV-related illnesses. HIV burden is unequally distributed globally and is most prevalent in the WHO African Region, accounting for over two-thirds of the people living with HIV (PLHIV) (WHO, 2023).

Approximately 12 million people held in prisons worldwide have been disproportionally affected by HIV and other blood-borne viruses despite the decline in the rate of new infections of HIV worldwide (Heidari *et al.*, 2015; UNODC, 2021). Contextual factors such as inadequate infection prevention and control measures, late case detection, absence of harm reduction measures, preventive therapy and timely treatment, poor nutrition, limited access to water, overcrowding and risky behaviours including alcohol or drug use (Werb *et al.*, 2008; Jürgens *et al.*, 2009) contribute to the increased transmission rates in prisons. The spread of HIV in prisons has significant public health implications as those employed in prisons, including guards, health workers, allied health staff and administrative workers are at risk of exposure to HIV through factors such as violence from incarcerated individuals or other workplace-related hazards (Sayyah *et al.*, 2019). Previous research revealed that the incarcerated, particularly those living with HIV, experience significant prejudice and discrimination, leading to isolation and unwillingness to disclose their status (Belenko *et al.*, 2016). Accessing HIV care and treatment within prison settings is also challenging due to limited resources, inadequate health-care services and insufficient education (Blue *et al.*, 2022), impacting treatment outcomes and general health of both incarcerated individuals and prisons officers.

Furthermore, incarcerated individuals living with HIV (ILHIV) who have interrupted their treatment may return to their communities without sufficient viral suppression (high viraemia) and facilitate the spread of HIV infection to the general population. Additionally, they may present with untreated or poorly treated disease, high viral loads and advanced HIV infection (Althoff *et al.*, 2013; Montague *et al.*, 2016; Vagenas *et al.*, 2016). In Ghana, the Prisons AIDS Control Programme was established in 2001 in response to the global trend of high vulnerability to HIV infection in prison services with respect to both officers and incarcerated individuals. HIV management in Ghanaian prisons involves a multifaceted approach. These include testing and counselling before incarceration, awareness creation and education among prison officers and incarcerated individuals, access to ART, reducing stigma against ILHIV and linkage to care after exit (Ghana Prisons Service, 2010). While significant efforts have been made to provide HIV services to prison incarcerated individuals in Ghana, this has been hampered by unique challenges such as financial and institutional constraints (Baffoe-Bonnie *et al.*, 2019). A recent assessment of the HIV situation conducted in the Ghana Prisons Service (GPS) reported an HIV prevalence of 1.7% among prison incarcerated individuals, comparable to the national prevalence of 1.67% (Ghana AIDS Commission 2020, 2021a).

The National HIV and AIDS Strategic Plan 2021–2025 has considered incarcerated individuals as a vulnerable or at-risk group, although they are not classified as a key population in Ghana. The programme acknowledges that the prison community requires attention and targeted HIV intervention to reduce the rate of new infections while minimising morbidity and mortality in Ghana (Ghana AIDS Commission, 2021b). To ensure Ghana achieves the Joint United Nations Programme on HIV/AIDS (UNAIDS) 95-95-95 targets of eliminating HIV/acquired immunodeficiency syndrome (AIDS) by 2030, there is the need to focus and align country-specific elimination programmes to ensure that no at-risk group or key population is left behind. As such, it is important to investigate HIV care in prisons from the perspective of those who receive this care. Hence, this study explored the experiences of ILHIV and on antiretroviral therapy (ART) in selected prisons in Ghana.

## Methods

### Study design and population

This study used a qualitative explorative descriptive design. It involved in-depth interviews of purposively selected ILHIV. The eligibility criteria included: adults living with HIV in prison for more than three months and having accessed ART services while incarcerated. Nonconsenting individuals, incarcerated individuals in juvenile detention and maximum-security prisons and incarcerated individuals incarcerated for less than three months were excluded from the study.

### Study sites selection and sampling

Purposive sampling was used in selecting the study site and participants. To begin, prisons with confirmed ILHIV per prison records were eligible to be sampled. This excluded prison establishments in the northern zone. The sampling was thus conducted in prisons sited in the Southern and Middle zones of Ghana. A list of medium security establishment was made, as per the exclusion criteria. Thereafter, to ensure sampling from a wide range of inmates that had similar characteristics of inmates across the country, maximum variation sampling was applied to select prison complexes with both male and female inmates. The selected prisons included the Ankaful Main Camp, Ankaful Contagious Disease Prison (CDP) and the prison complexes in Nsawam and Kumasi. The Ankaful Main Camp and CDP are located at Ankaful Prisons Complex in the Central Region of Ghana. The Main Camp Prison has a total of 617 incarcerated individuals, whereas the CDP accommodates a total inmate population of 61. The Nsawam Medium Security Prison is located in the Nsawam township along the Accra-Nsawam trunk road. The prison has approximately 3,500 incarcerated individuals. The Kumasi Prison complex is situated in Kumasi, the capital of the Ashanti Region of Ghana. The prison currently accommodates 1,985 incarcerated individuals (Ghana Prisons Service, 2022). This selection was necessary to ensure that the experiences of various categories of prisoners living with HIV was captured. Using the nurses in the infirmary as gatekeepers who had access to the health records of the inmates, eligible individuals were invited into the study. After initial discussions and clarifications, 16 participants who met the inclusion criteria and who consented were recruited into the study.

### Data collection methods

An interview guide (Supplementary 1) was used to collect data from participants via face-to-face semi-structured interviews. The interviews were guided by the HIV/AIDS Prevention, Care, Treatment and Support in Prison Settings: A Framework for an Effective National Response document (WHO and UNAIDS, 2006) and the United Nations Office on Drugs and Crime (UNODC) toolkit for HIV situation and needs assessment in Prisons (UNODC and European Monitoring Centre for Drugs and Drug Addiction, 2010) was used as a guide to unearth experiences in relation to general health-care services, HIV transmission, HIV/STI testing and counselling, HIV ART, support and referral and HIV stigma. The guides were pretested, and revisions were made based on feedback from experts and officers of the GPS.

The interviews were conducted by the researchers and trained staff in secured rooms made available by the prison authority per their guidelines. The GPS staff and research team received a one-day training about the interview guide content and also guidelines on how to engage and interact with incarcerated persons. During the interview, the doors were closed and only the participant, interviewer, notetaker were present. This process continued until saturation was reached after the 13th interview and confirmed with three additional interviews. The interviews lasted between 20 and 30 min. A total of 16 interviews were conducted based on the qualitative principle of saturation (Fusch and Ness, 2015).

### Ethical consideration

Ethical clearance was sought from GHS/ERC Review by the Ghana Health Service Ethical Committee (GHS-ERC: 011/09/22) and permission to conduct the study was obtained from the relevant authorities and study facilities. All COVID-19-related protocols were strictly observed throughout the processes to ensure the safety of the field team and participants. Written and oral informed consent was obtained from all participants. Participation was completely voluntary, and questions were constructed to be respectful and clear to participants. In respect of prison protocol, detailed sociodemographic characteristics were not collected from participants; however, each interview was assigned an identity number to allow for study purposes.

### Data analysis

In-depth interviews were audio recorded, transcribed and translated. Transcripts in Twi and Fante were first translated into English and then back to the original languages. Thematic analysis following the approach by Braun and Clarke (Braun and Clarke, 2006) using the ATLAS.Ti software version 8.4.4 was performed. To begin with, the researchers familiarised themselves with the data. In the process, the interviews were initially open coded by identifying recurring ideas, experiences and phenomena that were assigned labels or codes. Following this, the codes were categorised, and patterns were developed into themes and sub-themes. The analysis was iterative, with themes developed from the data and refined as the analysis progressed.

## Findings

### Summary characteristics of participants

As indicated earlier, 16 PLHIV in prison participated in the study. General information about the clients gleaned from the interview information indicates that majority of the participants were males (10, 62.5%). Two of the participants were on remand pending trial while the remaining were serving prison sentences. All but five (31.3%) of the participants tested positive for HIV for the first time in prison. All participants were receiving ART.

## Emergent themes

Three themes were generated from the analysis: *waking up to a positive HIV status*; *living with HIV a day at a time*; and *being my brother’s keeper: preventing HIV transmission*. Subcategories were generated under each major concept (Table 1).

## Theme one: Waking up to positive human immunodeficiency virus status

### Screening for human immunodeficiency virus/TB in prison

Most of the participants revealed undergoing screening for HIV/TB after arriving in the prison. The test was reportedly free for the incarcerated individuals:

It [HIV/TB test] was free […] we [incarcerated individuals] didn’t pay anything. [P1]

The narratives also revealed that following an initial positive HIV test in the prison, confirmatory tests were conducted in a public health facility as per national protocols:

After testing me here in prison, we want to outside hospital at K [teaching hospital]. At K too, they also tested me. Afterwards they told me I have HIV. We came back to prison yard and started giving me medicine. [P5]

It was evident that several of the participants were aware of their HIV status prior to their incarceration and therefore were not surprised when they tested positive during the screening. But for those who did not know their status, their initial reaction was fear:

I was afraid all I knew was that my world was coming to an end because I was going to die. [P2]

### Is the “counselling” in human immunodeficiency virus counselling and testing done in prison?

From the narratives, several of the participants did not receive pre-test information prior to the screening for HIV. One participant indicated not being prepared for the test psychologically. He said:

I didn’t go through any process, but I was in a different camp and there I was sent to the hospital for testing […] When the authorities in the prison sent me to the hospital for the test, the woman just asked me if I have heard about the illness and I said yes, that was all [P3]

All participants who indicated being screened reported that their consent was sought, and that the test was not under duress:

Here in prison, they ask you before they test you. [P6] They pampered us to allow ourselves to be tested they pampered us and did not treat us bad. [P9]

Although, consent was sought prior to the HIV screening, some participants indicated their initial unwillingness to undergo the testing. This attitude was fostered by the incarcerated individuals concern of the consequences of a positive HIV/TB diagnosis on their stay in the prison:

When they told me to do the test, I was not even willing to do because I did not know its consequences. [P4]

Some participants however reported positive experiences during the post-test counselling. The positive attitudes of the prison officers who undertook the post-test counselling resonated throughout the narratives:

I observe that they were patient in the counseling they offered because this sickness is not easy. [P1]

### The end or new beginning?

For some participants, the counselling addressed their concerns about their HIV diagnosis causing their death and brought a ray of hope by increasing their awareness of medications that could manage the conditions:

They [prison officers] counselled me about the disease [HIV] and told me the virus does not kill but when you continuously take your medication, you will be fine. [P3]

They advised me that this sickness cannot kill you […] If you continue to take your medication you will get the age God has ordained for you before you die. [P1]

Several of the participants said they received comfort and support from the officers. For most of them, the acceptance and non-judgmental posture of the prison officers was reassuring and refreshing:

You know when I was out there [home], I don’t have err HIV.

But since I came here, I have tested me positive. I know that I have HIV. The officers, they try their possible best to make sure that they take care of us […] Wherever we are, and they see that we are down because of our situation, they normally come closer to us and talk to us. [P11]

Others also received both emotional and financial support from the officers that gave them a new lease on life:

If it had not been for Mr XY [prison officer], I would have died at the time […] I was worrying and I couldn’t eat and do anything it was

Mr XY [officer] who will send them to buy food for me and call me to his office and advise me. But for now, I am alright [P8]

For most of the participants, the post-test counselling and the initiation on ART was the ray of hope that followed the diagnosis:

I was panicked because I thought that was the end of my life but they were patience to sit me down and counsel me before they gave me the drugs. It was that day that I realized when you continue taking your drugs everything will be fine. [P3]

## Theme two: Living with human immunodeficiency virus a day at a time

### Accessing antiretroviral therapy in prison

The narratives revealed that the conduit of accessing ART while incarcerated was dependent on incarcerated individuals’ status; whether serving a sentence or on remand pending trial. For those on remand the officers of the Criminal Investigator Department (CID) who were processing the cases were the main conduit for accessing ART:

If you say you are in remand, they will tell you to see your CID

[Criminal Investigation Department’s officer] to take your drugs. So, we really need [the] CID’s help. [P4]

For those who were serving a prison sentence, the main source for ART was the doctors who worked in the prison infirmary:

We get the medicine through the doctor that comes here. [P7]

Other participants also reported some changes in the processes through which they acquired their ART. These changes, some participants suggested, made the prison officers their source of ART:

At first, we used to go to the hospital for the drugs but after some time we will be there in the prison yard and the medicine [ART] will be given to us. [P5]

The narratives revealed different strategies of regulating ART dispensed across various prisons. According to several of the participants, they were often supplied quantities of ART drugs that lasted for a maximum of three months:

The drugs [ART] are there [infirmary] and they give us for 3 months. [P3]

Some participants also said they were only given an ART that could last for one week to keep on their person while the prison officers kept the rest of their stock and dispensed as and when the need arose:

So, when I come the madam [officer] give me the one that will take one week and when I finish, I go for some too. [P5]

For other participants also, all the ART was kept by the prison officers who administered it daily to them. Several participants opined that this strategy was not conducive as it affected their mealtimes and should therefore be reviewed:

After checking they sometimes give us drugs and the madam [officer] will keep it and be giving to us in the morning. [P6]

I wish they could give us [incarcerated individuals] the medicine to keep so that by the time the food is ready we will be able to eat it hot. But if it is with them, they give it to us at their own time. And by the time they give the medicine; our food will be cold. [P3]

### The attempts and struggles of remaining anonymous

Participants reported that efforts were made by the officers to maintain confidentiality of the incarcerated individuals HIV positive status:

As for here [prison], they [officers] keep it [HIV status] secret. They don’t tell incarcerated individuals about your status. [P6]

However, some participants ascribed breach of confidentiality to other incarcerated individuals who were assigned to work in the infirmary and so had access to patients’ medical records. While the other incarcerated individuals may be offering some useful services to officers in the infirmary, their presence in the facility is a source of concerns about confidentiality for ILHIV:

If you have observed some of the incarcerated individuals help the officers in the hospital here [infirmary]. So, they take our information to the other incarcerated individuals […] I don’t believe that the officers will tell the incarcerated individuals that certain incarcerated individuals have these sicknesses [HIV] because when we come here it is the incarcerated individuals who give us our medication and they are the people who take our information into the yard. [P8]

To further promote confidentiality and combat HIV-related stigma and discrimination, emerging evidence indicated promotion of non-disclosure of HIV status to other incarcerated individuals:

They [officers], pamper you and tell you not to tell anyone you are HIV positive. [P4]

Some participants said that the unintended disclosure of their HIV status occurred in the prisons. It was evident that, other incarcerated individuals’ awareness of someone’s HIV status had negative repercussions such as gossiping, which set them up for stigmatisation and discrimination:

I did not tell him anything [HIV status] […] He has been seeing me taking drugs often […] If your friends find out, you are HIV positive they do tell other people. [P5] We are pleading, if they want to take us to outside hospital, they should hide us small, and it shouldn’t be open. Even that one can make you think a lot and can kill you fast. [P7]

### The bane of human immunodeficiency virus–related stigma and discrimination

Some participants indicated they had never experienced HIV-related stigma and discrimination in prison. This was mainly attributed to the efforts of the prison officers:

There is no discrimination in this house because of the good work the officers are doing in this prison. [P1]

Few participants, on the other hand, reported being stigmatised in prison as a result of their HIV diagnosis. The act of stigmatisation was reportedly perpetuated by other incarcerated individuals:

No officer has done [stigma and discrimination] that but some of my friends use it to frighten me. [P4] People [incarcerated individuals] were pointing accusing fingers at me so I was always disturbed and became worried. [P8]

Some participants shared experiences of being labelled as a result of their HIV diagnosis:

For the incarcerated individuals if they hear you have this illness […] they will also point accusing fingers at you like when you are going to take your medication in the morning when they see you, they make statements like this are the are sick people when this happens, it makes us feel uneasy. [P8]

One participant shared an experience of enacted verbal stigmatisation in prison as a result of his HIV diagnosis. He said:

Someone was owing me, and I said I was going to take it back. He told me that, when I go for the money, I will not even use it for anything because I have a very serious illness [HIV] and I will use the money to buy drugs. [P4]

### The compounded needs of being incarcerated and living with human immunodeficiency virus

Narratives revealed that participants struggled with their daily upkeep as they took their ART to maintain their health. Most of them reported a lack or adequacy in the provision of food which affected their adherence to the ART:

Here they will give the drugs, but feeding is a problem. I have been given a medicine since morning but no food. So that is the problem we are facing here. You need to eat when given a medicine but here is not like that. [P5]

Other participants opined that improving incarcerated individuals’ nutrition while on ART will also avert exacerbation of their illness and improve their appearance thereby reducing the likelihood of stigma and discrimination:

What we need is the food and drugs. If you don’t eat well and you grow lean that incarcerated individuals can discriminate against you but if we eat well and we gain strength it will reduce […] Those [incarcerated individuals] saying and pointing fingers at you that you are HIV positive will stop because I believe when you eat well and you grow from strength to strength; they will not know your status. [P7]

Other participants also suggested enacting rules against stigma and discrimination in prison to curb its occurrence:

I want them to make a rule not to stigmatized or discriminate those with the disease. This will help to eliminate or reduce it. [P4]

## Theme three: being my brother’s keeper: preventing human immunodeficiency virus transmission

### Taking extra precaution

Several of the participants indicated efforts they made to prevent the transmission of the virus to other incarcerated individuals, although these efforts are not always welcomed by other incarcerated individuals:

Those of us who have tested and received counselling, we know how we can manage ourselves for when I am going to barber my hair, I take my own blade and don’t allow the barber to use his machine. I also use my own comb, I don’t allow them to use anybody’s comb; I take my own comb. Some people will complain that I am too overprotective, but I know why I am doing that. [P8]

These precautionary measures to prevent transmission were sometimes unwelcomed as a result of the cost of acquiring such items such as blades in the prison:

[…] blade is even expensive so anybody who sees used blade can also use to trim his fingers […] but the problem is the monetary issues and we need help. [P5]

### Promoting knowledge through health education

The narratives revealed consistent efforts of curbing transmission of HIV in prison through health education. Participants across prisons, reported scheduled sessions for health education on HIV:

We do it [education] 4 times in a month and once in every week. The officers come and meet our in-charge here in our cell to educate us.

Sometimes some incarcerated individuals go outside but when they come, we all come together and they will educate us on the diseases. [P3]

The inclusion of incarcerated individuals as educators was reported by all the participants and reportedly improved the incarcerated individuals’ knowledge.

## Discussion

This study explored the experiences of incarcerated persons living with HIV and accessing ART care in Ghanaian prisons. It was evident that participants underwent HIV screening in the prisons. Consequently, several incarcerated persons became aware of their HIV positive status in prison. This finding is congruent with studies conducted in other countries (Nelwan *et al.*, 2016; Tavakoli *et al.*, 2022). Evident in this study was the fact that, the offer for HIV testing was provider-initiated and was without compulsion which is an acceptable strategy for HIV testing in Ghana (Ghana AIDS Commission, 2013). This finding is important, as it points to the fact that HIV testing in prison generally follows ethical considerations as outlined by the WHO and other global organisations (UNODC and European Monitoring Centre for Drugs and Drug Addiction, 2010; UNODC, UNAIDS and WHO Regional Office for Europe, 2010).

From the experiences of the ILHIV, it was also evident that the prisons also followed protocol by ensuring confirmatory tests were conducted for those incarcerated individuals who tested positive to HIV. Experts recommend confirmatory HIV tests as it ensures reduction in false-positive results, increases accuracy of HIV tests results (World Health Organization, 2020) as well as contributes to acceptance of HIV positive diagnosis among different populations (Scognamiglio *et al.*, 2018; Abraham and Clow, 2022).

In this study, an HIV positive test was an important milestone as it represented the beginning of a counselling relationship between the officers and the ILHIV which was marked with patience, concern and support from the officers. Fuge *et al.* (2022) reported that social support from officers was an important facilitator for adherence to ART among incarcerated ILHIV and provided a sense of safety for them as they knew the officers were available to attend to their concerns when the need arose.

From the participants shared experiences, ART was accessible in the prison. This findings corroborates results reported in Malawi (Gondwe *et al.*, 2021). ART access was attributed to the presence of well-established on-site infirmaries where health professionals provided services. However, ILHIV who were on remand and awaiting trial shared concerns about their access to ART as the responsibility for refill was left to the CIDs who were not part of prison staff and were not on site. It is, therefore, necessary to address this lapse, as lack of access to ART by a section of the population had implications on their health as well as the general efforts of the prisons to reduce infection rate among incarcerated individuals.

Furthermore, incarcerated individuals’ indicated struggles to maintain anonymity and confidentiality of their HIV status in prison. This was mostly attributed to prison protocols and procedures that undermined maintenance of anonymity of clients on ART. For instance, the context-specific arrangements on ART dispensing to the clients daily at the infirmary and only ILHIV having to visit the infirmary for ART daily or as scheduled by the prisons protocols was of concern to several clients. These arrangements illuminated the lack of privacy for ILHIV in prisons especially when accessing ART services. This finding agrees with findings from other jurisdictions (Small *et al.*, 2009; Esposito, 2012; Chimoyi *et al.*, 2021). For some participants, the approach had negative implications as it created anxiety and hypervigilance among ILHIV in prisons for fear of unintended disclosure (Kemnitz *et al.*, 2017). This unintended outcome of implementation of policy should be considered when strategizing to improve ART access while regulating the quantity of medication in circulation in prisons.

Also, ILHIV were reportedly encouraged not to disclose their positive HIV status to other incarcerated individuals. This finding contradicts the national policy that encourages PLHIV to disclose status among others to break the cycle of discrimination and stigma and to garner support for treatment adherence (MOH/GHS, 2014). This strategy may be necessary to avert discrimination and stigmatisation in the confines of the prisons. In spite of the efforts of the ILHIV not to disclose and incarcerated individuals’ conviction in the officers to maintain their privacy, there were concerns of breaches in confidentiality accessing ART by other incarcerated individuals who worked in prison infirmaries, who had access to medical records or the opportunity to observe clients collect their medications. Although, this has not been reported by other studies, it is important to ensure that prison management educate incarcerated individuals and workers on consequences of breach of confidentiality and also establish pathways for reporting such incidences as indicated in the HIV/TB Workplace Policy and Implementation Strategy (Ghana Prisons Service, 2010).

Contrary to reports from Namibia and other jurisdictions where ILHIV on ART were served special meals with additional protein-rich foods (Shalihu *et al.*, 2014; Chimoyi *et al.*, 2021), there were no indications of such privileges in the Ghanaian prisons. Participants experiences of insufficient nutritional intake in this study has also been reported in some Ghanaian prisons (Lokpo *et al.*, 2020). Insufficient food security and poor nutrition may compromise adherence to and responsiveness to ART, increase the development of AIDS-related illnesses and worsen the socioeconomic effects of the virus (Berhe *et al.*, 2013) and thus, should be of course to the prison authorities. The additional benefits of a well-nourished ILHIV on averting suspicion and stigmatisation was stressed by participants in this study as they perceived that looking malnourished was a precursor for being suspected of being HIV positive.

The ILHIV perceived that prison officers did not stigmatise or discriminate against clients. This finding is congruent with those reported in the USA (Belenko *et al.*, 2016). This laudable findings could be associated to the efforts by prison management to enhance the knowledge of officers on HIV, as well as the documented policy on consequences of stigma and discrimination against incarcerated individuals PLHIV by officers (Ghana Prisons Service, 2010). There were, however, reports of stigma perpetuated among the incarcerated individuals similar to findings from South Africa (Chimoyi *et al.*, 2021). We agree with findings from Chimoyi *et al.* that being sensitive to HIV-related stigma in prisons is essential as this may not only inform HIV-related service delivery in prisons but will also empower ILHIV to take measures to break the power held by others over them because of their conditions. Consequently, the successes being chalked with reducing HIV-related stigma in the general population could be translated into the prisons.

## Strengths and limitations of the study

The study is novel as there is currently limited study in Ghana on the experiences of ILHIV in prisons. Also, the engagement of prison officers in shaping the interview guide and participant recruitment to an extent that was ethically acceptable, offered insights that may have been omitted if they were not included. However, the researchers acknowledge the possibility of selection bias with the use of gatekeepers in recruitment which could result in the exclusion of certain individuals from participating. To address this, the researchers ensured the study sample was drawn from various prisons and heterogenous groups to ensure diverse perspectives. Also, the possibility of power dynamics and coercion to participate was also anticipated. It was therefore ensured that all participants were informed of their autonomous right to decline participation or stop the interviews at any time without any effect on their incarceration.

Again, social desirability underscoring the answers of participants cannot be discounted, as they would continue to receive care from the same officers who approached them about the study and also continue to serve their sentences. This was however reduced by ensuring that those officers where not physically present during the interviews.

## Conclusion

This study provided valuable insights into the experiences of PLHIV in Ghanaian prisons. HIV testing and counselling services provided in the prisons were without coercion. Although several strategies are implemented to ensure access to ART for the ILHIV, the approach is not without it challenges, such as fear of unintended disclosure that presents the opportunity of stigmatisation and discrimination among incarcerated individuals. Furthermore, the need to enhance the nutritional intake for PLHIV on ART was also evident. This will not only improve ART adherence but will also ensure that prison ILHIV will have better quality of life.

## Acknowledgements

The authors acknowledge the efforts of the Management of the GPS, for their immerse support for this project. The authors also express gratitude to the participants for sharing their experiences with the authors.

*Funding:* This project was funded using Global Funds money through the Ghana Health Services, National HIV/AIDS Control Programme.

*Data availability:* The data set from which the findings of the study emerged is available upon reasonable request to the corresponding author.

Conflict of interest: The authors declare no conflict of interest.

*Authors’ contributions:* Dorcas Obiri-Yeboah, Susanna Aba Abraham, Yvonne Ayerki Nartey, Elizabeth Agyare, Stephen Ayisi Addo and Francis Annor designed the research and revised the manuscript. Dorcas Obiri-Yeboah, Benedict Osei Tawiah, Matilda Nyampong, Marijanatu Abdulai. Kwadwo Koduah Owusu and Obed Cudjoe were involved in data collection. Susanna Aba Abraham, Dorcas Obiri-Yeboah and Benedict Osei Tawiah were involved in data analysis. Obed Cudjoe, Yvonne Ayerki Nartey and Susanna Aba Abraham drafted the manuscript. All authors revised and approved the final manuscript.

## Supplementary Material



## Figures and Tables

**Table 1:** Major themes and subthemes

Themes	Sub-theme
*One* Waking up to a positive HIV status	Screening for HIV/TB in prison Is the “counselling” in HIV counselling and testing done in prison? The end or a new beginning?
*Two* Living with HIV a day at a time	Accessing ART in prison The bane of HIV-related stigma and discrimination The attempts and struggles of remaining anonymous The compounded needs of being incarcerated and living with HIV
*Three* Being my brother’s keeper: preventing HIV transmission	Taking extra precaution Promoting knowledge through health education

**Source:** Table by authors

## Supplementary material

The supplementary material for this article can be found online.

## References

[ref001] Abraham, S.A. and Clow, S.E. (2022), “HIV counselling and testing experiences of expectant mothers in the prevention of vertical transmission programme: implications for policy and service delivery”, Therapeutic Advances in Infectious Disease, Vol. 9 No. X, pp. 1-10, doi: 10.1177/20499361221078424. PMC8859687 35198199

[ref002] Althoff, A.L., *et al*. (2013), “Correlates of retention in HIV care after release from jail: results from a multi-site study”, AIDS and Behavior, Vol. 17 No. S2, pp. 156-170, doi: 10.1007/s10461-012-0372-1. PMC3714328 23161210

[ref003] Baffoe-Bonnie, T., *et al*. (2019), “Access to a quality healthcare among prisoners – perspectives of health providers of a prison infirmary, Ghana”, International Journal of Prisoner Health, Vol. 15 No. 4, pp. 349-365, doi: 10.1108/IJPH-02-2019-0014. 31532341

[ref004] Belenko, S., *et al*. (2016), “HIV stigma in prisons and jails: results from a staff survey”, AIDS and Behavior, Vol. 20 No. 1, pp. 71-84, doi: 10.1007/s10461-015-1098-7. 26036464 PMC4669236

[ref005] Berhe, N., Tegabu, D. and Alemayehu, M. (2013), “Effect of nutritional factors on adherence to antiretroviral therapy among HIV-infected adults: a case control study in Northern Ethiopia”, BMC Infectious Diseases. BMC Infectious Diseases, Vol. 13 No. 1, p. 1, doi: 10.1186/1471-2334-13-233. 23701864 PMC3669031

[ref006] Blue, C., Buchbinder, M., Brown, M.E., Bradley-Bull, S. and Rosen, D.L. (2022), “Access to HIV care in jails: perspectives from people living with HIV in North Carolina”, Plos One, Vol. 17 No. 1, p. e0262882.doi, doi: 10.1371/journal.pone.0262882. 35073350 PMC8786150

[ref007] Braun, V. and Clarke, V. (2006), “Using thematic analysis in psychology”, Qualitative Research in Psychology, Vol. 3 No. 2, pp. 77-101, doi: 10.1191/1478088706qp063oa.

[ref008] CDC (2023), “Leading progress in ending TB, global health and TB”, available at: www.cdc.gov/globalhivtb/who-we-are/about-us/globaltb/globaltb.html (accessed 12 May 2023).

[ref009] Chimoyi, L., *et al*. (2021), “Erratum: HIV-related stigma and uptake of antiretroviral treatment among incarcerated individuals living with HIV/AIDS in South African correctional settings: a mixed methods analysis”, (PLoS ONE (2021) 16:, Vol. 16 No. 7, p. e0254975, doi: 10.1371/journal.pone.0259616. PMC8323907 34329311

[ref010] Esposito, M. (2012), “«Double burden»: a qualitative study of HIV positive prisoners in Italy”, International Journal of Prisoner Health, Vol. 8 No. 1, pp. 35-44, doi: 10.1108/17449201211268273. 25757860

[ref011] Fuge, T.G., Tsourtos, G. and Miller, E.R. (2022), “Factors affecting optimal adherence to antiretroviral therapy and viral suppression amongst HIV-infected prisoners in South Ethiopia: a comparative cross-sectional study”, AIDS Research and Therapy, Vol. 19 No. 1, pp. 1-14, doi: 10.1186/s12981-022-00429-4. 35093100 PMC8800260

[ref012] Fusch, P.I. and Ness, L.R. (2015), “Are we there yet? Data saturation in qualitative research”, The Qualitative Report, Vol. 20 No. 9, pp. 1408-1416, doi: 10.46743/2160-3715/2015.2281.

[ref013] Ghana AIDS Commission (2013), “National HIV&AIDS, STI policy.pdf”, Accra: GAC, pp. 1-67, available at: www.policyproject.com/pubs/policyplan/GHA_AIDS_Policy.pdf

[ref014] Ghana AIDS Commission (2020), National HIV & AIDS Strategic Plan 2021–2025, Accra, available at: www.ghanaids.gov.gh/mcadmin/Uploads/GAC%20NSP%202021-2025%20Final%20PDF.pdf

[ref015] Ghana AIDS Commission (2021a), HIV and Social Protection Evidence for Policy and Actionon Hiv and Social Protection Ghana Aids Commission, Accra, available at: www.ghanaids.gov.gh/mcadmin/Uploads/HIVANDSOCIALPROTECTION.pdf

[ref016] Ghana AIDS Commission (2021b), “National HIV & Aids strategic plan 2021-2025”.

[ref017] Ghana Prisons Service (2010), “HIV/TB WORKPLACE POLICY & implementation strategy”, Policy. Accra, available at: www.ilo.org/wcmsp5/groups/public/–-ed_protect/–-protrav/–-ilo_aids/documents/legaldocument/wcms_191117.pdf

[ref018] Ghana Prisons Service (2022), “Prison establishment”, available at: https://ghanaprisons.gov.gh/ (accessed 3 August 2022).

[ref019] Gondwe, A., *et al*. (2021), “Prisoners’ access to HIV services in Southern Malawi: a cross-sectional mixed methods study”, BMC Public Health, Vol. 21 No. 1, pp. 1-10, doi: 10.1186/s12889-021-10870-1. 33910547 PMC8080321

[ref020] Heidari, T., *et al*. (2015), “Maternal experiences of their unborn child’s spiritual care patterns of abstinence in Iran”, Journal of Holistic Nursing, Vol. 33 No. 2, pp. 146-158, doi: 10.1177/0898010114551416. 25288610

[ref021] Jürgens, R., Ball, A. and Verster, A. (2009), “Interventions to reduce HIV transmission related to injecting drug use in prison”, The Lancet Infectious Diseases, Vol. 9 No. 1, pp. 57-66, doi: 10.1016/S1473-3099(08)70305-0. 19095196

[ref022] Kemnitz, R., *et al*. (2017), “Manifestations of HIV stigma and their impact on retention in care for people transitioning from prisons to communities”, Health and Justice. Health & Justice, Vol. 5 No. 1, pp. 1-11, doi: 10.1186/s40352-017-0054-1. 28589252 PMC5461223

[ref023] Lokpo, S.Y., *et al*. (2020), “Evaluation of dietary patterns and haematological profile of apparently healthy officers of the central prisons in the Ho municipality. A cross sectional study”, Scientific African, Vol. 7, p. e00284, doi: 10.1016/j.sciaf.2020.e00284.

[ref024] MOH/GHS (2014), National Guidelines for Prevention of Mother-to-Child Transmission of HIV, Ghana Publishing House, Accra.

[ref025] Montague, B.T., *et al*. (2016), “Systematic assessment of linkage to care for persons with HIV released from corrections facilities using existing datasets”, AIDS Patient Care and STDs, Vol. 30 No. 2, pp. 84-91, doi: 10.1089/apc.2015.0258. 26836237 PMC4753628

[ref026] Nelwan, E.J., *et al*. (2016), “Routine or targeted HIV screening of Indonesian prisoners”, International Journal of Prisoner Health, Vol. 12 No. 1, pp. 17-26, doi: 10.1108/IJPH-04-2015-0012. 26933989

[ref027] Sayyah, M., *et al*. (2019), “Global view of HIV prevalence in prisons: a systematic review and meta-analysis”, Iranian Journal of Public Health, Vol. 48 No. 2, pp. 217-226, doi: 10.18502/ijph.v48i2.816. 31205875 PMC6556176

[ref028] Scognamiglio, P., *et al*. (2018), “HIV rapid testing in community and outreach sites: results of a nationwide demonstration project in Italy”, BMC Public Health. BMC Public Health, Vol. 18 No. 1, pp. 1-9, doi: 10.1186/s12889-018-5680-6. PMC6006581 29914449

[ref029] Shalihu, N., *et al*. (2014), “Namibian prisoners describe barriers to HIV antiretroviral therapy adherence”, AIDS Care, Vol. 26 No. 8, pp. 968-975, doi: 10.1080/09540121.2014.880398. 24499371

[ref030] Small, W., *et al*. (2009), “The impact of incarceration upon adherence to HIV treatment among HIV-positive injection drug users: a qualitative study”, AIDS Care, Vol. 21 No. 6, pp. 708-714, doi: 10.1080/09540120802511869. 19806487

[ref031] Tavakoli, F., *et al*. (2022), “HIV testing among incarcerated people with a history of HIV-related high-risk behaviours in Iran: findings from three consecutive national bio-behavioural surveys”, BMC Infectious Diseases, Vol. 22 No. 1, pp. 1-9, doi: 10.1186/s12879-022-07897-z. 36471282 PMC9721074

[ref032] UNODC (2021), “Data matters, statistical activities, surveys and standards”, available at: www.unodc.org/unodc/en/data-and-analysis/data-matters.html (accessed 12 May 2023).

[ref033] UNODC and European Monitoring Centre for Drugs and Drug Addiction (2010), “HIV in prisons Situation and needs assessment toolkit”, *Vienna*, available at: www.unodc.org/documents/hiv-aids/publications/HIV_in_prisons_situation_and_needs_assessment_document.pdf

[ref034] UNODC, UNAIDS and WHO Regional Office for Europe (2010), “Policy brief HIV testing and counselling in prisons and other closed settings”, World Health. Vienna, Austria, available at: www.unodc.org/documents/hiv-aids/UNODC_WHO_UNAIDS_2009_Policy_brief_HIV_TC_in_prisons_ebook_ENG.pdf

[ref035] Vagenas, P., *et al*. (2016), “HIV-infected men who have sex with men, before and after release from jail: the impact of age and race, results from a multi-site study”, AIDS Care, Vol. 28 No. 1, pp. 22-31, doi: 10.1080/09540121.2015.1062464. 26275122 PMC4713253

[ref036] Werb, D., *et al*. (2008), “HIV risks associated with incarceration among injection drug users: implications for prison-based public health strategies”, Journal of Public Health, Vol. 30 No. 2, pp. 126-132, doi: 10.1093/pubmed/fdn021. 18387974

[ref037] WHO (2023), “HIV and AIDS”, Fact Sheet, available at: www.who.int/news-room/fact-sheets/detail/hiv-aids (accessed 12 May 2023).

[ref038] WHO and UNAIDS (2006), “HIV/AIDS prevention, care, treatment and support in prison settings. A framework for an effective national response”, New York, available at: http://data.unaids.org/pub/report/2006/20060701_hiv-aids_prisons_en.pdf

[ref039] World Health Organization (2020), “Consolidated guidelines on HIV testing services 2019, guidelines”, Geneva: WHO Press, available at: www.who.int/publications/i/item/978-92-4-155058-1

