# Supplementary material for: Unravelling the experiences of incarcerated individuals living with HIV on ART: a qualitative study in Ghanaian prisons
Source: Int J Prison Health (2024). 2024 Jan 19;20(2):186–99. doi: 10.1108/IJOPH-06-2023-0031 (PMC11342671; doi:10.1108/IJOPH-06-2023-0031)
Supplement: Supplementary file 1 [file intjprisonhealth-20-0186-s001.docx]

**Supplementary 1: Interview Guide for Inmates Living With HIV (Author’s own work)**

**DISCUSSION POINTS**

**General healthcare services**

- In your opinion, what standard of healthcare do inmates currently receive?
- Compared to your experiences outside the prison, can you compare the standard of healthcare in prison with that outside?
- In your opinion, what standard of healthcare should inmates receive?

**HIV Transmission**

- Can you tell me about some current measures being used to prevent transmission of HIV in the prison? (i. Transmission between inmates ii. Transmission between inmates and prison officers)
- Can you tell me about any measures in place to prevent mother to child transmission in the prison (for female inmates)?

**HIV Testing and Counselling**

- Can you tell me about how you were tested for HIV and what the process and your experience was like?
- Can you tell me about what counselling or advice you received before and after you were told you had HIV?
- Is there anything you think could have been done better for you in testing and counselling for HIV in the prison?

**HIV ART Therapy, Support and Referral**

- Can you tell me about how you receive your ART medication?
- Is there anything that you think can be done to improve how you get your ART medication and the support you receive in the prison?
- Can you tell me about a time that you were referred to a centre outside of the prison for HIV care?
- In your opinion, is there anything that could have been done better regarding your referral to the hospital or the follow-up after your referral?

**Other services**

- Do you receive any education on prevention of TB and other STIs?
- If so, what form does the education take and how often?
- Would you say you have enough opportunities have health related education?
- Concerning TB specifically, how often are you screened?
- How would you describe services available for TB screening, diagnosis, treatment and prevention?
- Have you ever had screening for any other STIs like hepatitis B? Are such screening services available and accessible to inmates?
- Do you have other conditions than HIV? Can you state it and how you receive health care for that?

**HIV Stigma**

- Can you tell me about your experiences with stigma and discrimination as an inmate living with HIV (i. From other inmates ii. From prison officers iii. From family)
- What are the current measures that you know of that have been put in place to tackle stigma and discrimination against inmates with HIV?
- What do you think needs to be done to prevent or reduce stigma against inmates living with HIV?

**HIV Funding and resources**

- What has been your experience with paying for HIV related services whilst you have been in prison?
- How would you describe current resources (including infrastructural and human) available to you for your care?
- What resources (including infrastructural and human) and funding do you think are needed to improve your HIV care in the prison setting?
